# Nuclear-import receptors as gatekeepers of pathological phase transitions in ALS/FTD

**DOI:** 10.1186/s13024-023-00698-1

**Published:** 2024-01-22

**Authors:** Bilal Khalil, Miriam Linsenmeier, Courtney L. Smith, James Shorter, Wilfried Rossoll

**Affiliations:** 1 Department of Neuroscience, Mayo Clinic, Jacksonville, FL 32224 U.S.A.; 2 Department of Biochemistry and Biophysics, Perelman School of Medicine, University of Pennsylvania, Philadelphia, PA 19104 U.S.A.; 3 Mayo Clinic Graduate School of Biomedical Sciences, Neuroscience Track, Mayo Clinic, Jacksonville, FL 32224 U.S.A.

**Keywords:** Amyotrophic lateral sclerosis, Frontotemporal dementia, FUS, TDP-43, Protein aggregation, Nucleocytoplasmic transport, Importin, Nuclear pore, Chaperone, Aberrant phase transition, RNA-binding proteins

## Abstract

Amyotrophic lateral sclerosis (ALS) and frontotemporal dementia (FTD) are fatal neurodegenerative disorders on a disease spectrum that are characterized by the cytoplasmic mislocalization and aberrant phase transitions of prion-like RNA-binding proteins (RBPs). The common accumulation of TAR DNA-binding protein-43 (TDP-43), fused in sarcoma (FUS), and other nuclear RBPs in detergent-insoluble aggregates in the cytoplasm of degenerating neurons in ALS/FTD is connected to nuclear pore dysfunction and other defects in the nucleocytoplasmic transport machinery. Recent advances suggest that beyond their canonical role in the nuclear import of protein cargoes, nuclear-import receptors (NIRs) can prevent and reverse aberrant phase transitions of TDP-43, FUS, and related prion-like RBPs and restore their nuclear localization and function. Here, we showcase the NIR family and how they recognize cargo, drive nuclear import, and chaperone prion-like RBPs linked to ALS/FTD. We also discuss the promise of enhancing NIR levels and developing potentiated NIR variants as therapeutic strategies for ALS/FTD and related neurodegenerative proteinopathies.

**Graphical Abstract:**

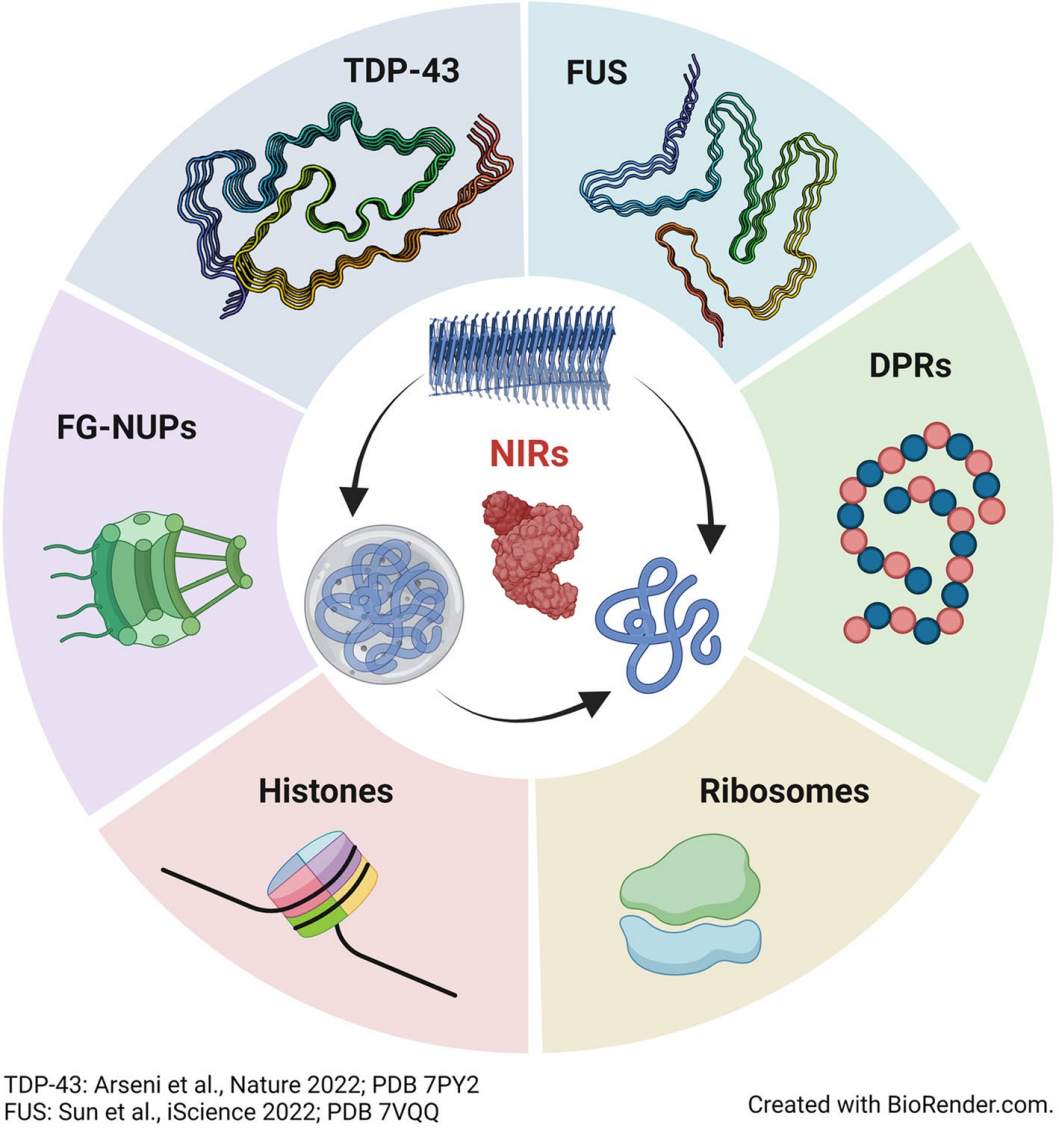

## Introduction

Protein mislocalization and aberrant phase transitions are powerful drivers of pathology in late-onset neurodegenerative diseases [1, 2]. Two fatal neurodegenerative disorders, amyotrophic lateral sclerosis (ALS) and frontotemporal dementia (FTD), reside at opposite ends of a continuum of disease states that share underlying genetics, clinical features, and the characteristic accumulation of TAR DNA-binding protein-43 (TDP-43) or other nuclear RNA-binding proteins (RBPs) in detergent-insoluble aggregates in the cytoplasm [3]. Disease-causing mutations in several genes encoding RBPs, including TDP-43, members of the FET protein family fused in sarcoma (FUS), Ewing's sarcoma protein (EWSR1), and TATA-binding protein-associated factor 15 (TAF15), and heterogenous nuclear ribonucleoproteins hnRNPA1 and hnRNPA2, suggest a direct role for RBP mislocalization and aggregation in the disease process [4–7]. The relationship between the physiological phase separation of these RBPs into dynamic condensates, often mediated by their intrinsically disordered prion-like domains (PrLDs), and the pathological formation of stable and deleterious amyloids in neurodegenerative diseases is an area of intense investigation.

The pathological accumulation of nuclear RBPs in cytoplasmic aggregates suggests a connection between protein mislocalization and aberrant phase transitions that is further supported by the observation of nucleocytoplasmic transport defects in a growing number of neurodegenerative disorders [8–11]. Studies have converged on the finding that nuclear-import receptors (NIRs) act as gatekeepers that preserve the proper nuclear localization of disease-associated RBPs, while preventing and even reversing aberrant phase transitions into pathological aggregates [2, 12–18]. This heretofore underappreciated chaperone and disaggregase activity of NIRs can reduce the cytoplasmic aggregation of prion-like RBPs. The canonical nuclear import activity of NIRs then enables restoration of prion-like RBPs to the nucleus, which reinstates their nuclear function and mitigates neurodegeneration. In this review, we discuss the canonical transport functions of NIRs, evidence of their disruption in disease, and how they can be harnessed to therapeutically target pathological RBP aggregation.

## Nuclear-transport proteins associate with cargo proteins and FG-rich Nucleoporins to regulate nucleocytoplasmic transport

### The karyopherin family of nucleocytoplasmic transport receptors

Nucleocytoplasmic transport (NCT) of proteins and RNA is critical to maintain proper subcellular compartmentalization in eukaryotes. This efficient and highly regulated process depends on three components: 1) a selective gateway for large macromolecules formed by the assembly of large multi-protein nuclear pore complexes (NPCs); 2) a family of nuclear-transport receptor proteins (NTRs) that facilitate the NCT of their respective cargo through the NPC; and 3) a concentration gradient of nuclear GTP-bound and cytoplasmic GDP-bound Ras-related nuclear protein (Ran) that confers directionality to the transport processes by regulating compartment-specific cargo binding and release [19]. NPCs are eightfold symmetrical cylindrical assemblies that are embedded in the nuclear envelope and are composed of multiple copies of ~ 30 different proteins called nucleoporins (Nups) [20]. There has been tremendous progress in understanding the architecture of the vertebrate NPC and its subcomplexes based on cryo-EM and artificial intelligence-based structure modeling that inform aspects of NPC biogenesis, function, and regulation [21–26]. Within the central channel of the NPC scaffold, a family of Nups harboring intrinsically disordered phenylalanine-glycine repeats (FG-Nups) form a densely packed network, which may have properties akin to a hydrogel or viscoelastic network fluids [27–30]. It should be noted, however, that FG-domain hydrogels formed in solution may be an imperfect model of the NPC permeability barrier: anchoring of a distinct number of FG-Nups with three-dimensional precision inside the NPC scaffold in situ changes their properties as compared to phase-separated FG-Nups in vitro [31]. Other studies propose that the permeability barrier of the NPC is better described as a meshwork of polymer brushes that is organized by the NPC scaffold, and not a phase-separated condensate [32]. This network of FG-Nups does not form a firm size threshold, but increasingly restricts macromolecules to enter or exit the nucleus via passive diffusion based on their size [33, 34]. Ions, nucleotides, salts, and proteins below ~ 40 kDa can passively transit through the nuclear pore, whereas the transport of larger macromolecular protein and RNA cargo depends on the diverse but structurally related karyopherin family of NTRs.

**Fig. 1:**
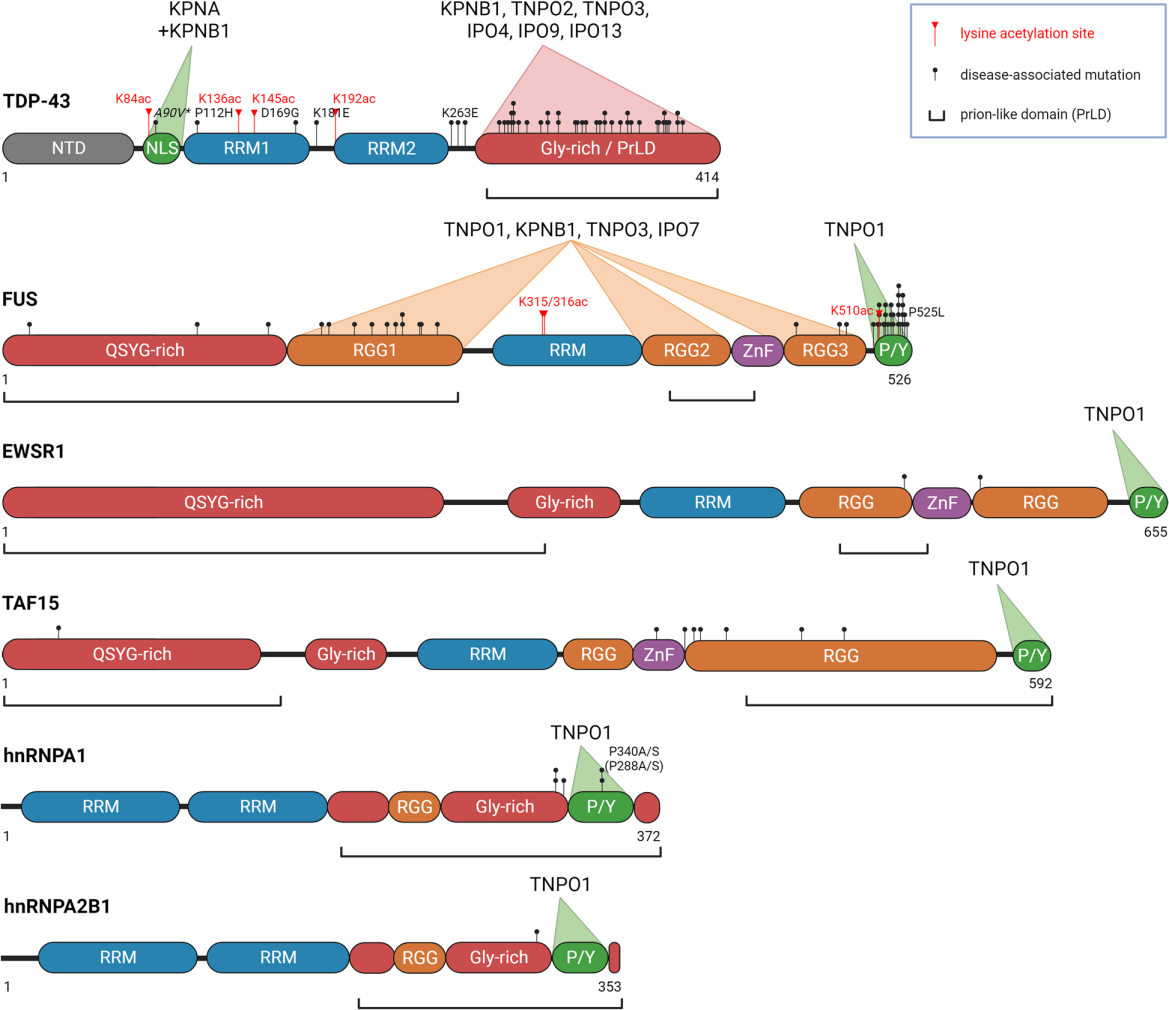
Schematic of TDP-43, FET proteins, hnRNPA1 and hnRNPA2 domains and their interaction with importins. To regulate nuclear import and phase transition of RBPs, TNPO1 binds the PY-NLS of FUS and, with a lower affinity, its RGG domains, while KPNB1 interacts with both the NLS (via KPNA or importin-α) and PrLD of TDP-43. Of note, most ALS disease-causing mutations are located in the PY-NLS and PrLD of FUS and TDP-43, respectively. Lysine acetylation sites that regulate phase separation of these prion-like RBPs are also highlighted. Other importins also bind the RGG domains of FUS and PrLD of TDP-43. Thus far, only TNPO1 has been shown to bind the PY-NLS of EWSR1, TAF15, hnRNPA1 and hnRNPA2. The *A90V mutation in *TARDBP* is also found in the healthy population. Brackets indicate prion-like domains (PrLDs) as defined by their amino acid composition [63]. NLS = nuclear localization signal; NTD = N-terminal domain; P/Y = Pro-Tyr nuclear localization signal; PrLD = prion-like domain; QSYG-rich = Gln, Ser, Tyr, and Gly-rich domain; RGG = Arg-Gly-Gly repeat domain; RRM = RNA-recognition motif; ZnF = zinc finger domain. Created with BioRender.com

Karyopherins are subdivided into two subfamilies: importin-α and karyopherin-β proteins. Importin-α and karyopherin-β family members are highly flexible proteins that are composed of tandem α-helical ARM (armadillo) repeats or similar HEAT (Huntingtin, elongation factor 3, protein phosphatase 2A, and signaling kinase TOR1) repeats, respectively [35]. Importin-α proteins, also known as karyopherin-α proteins, are encoded by *KPNA1-7* in humans, and act as adaptor proteins that directly bind classical monopartite or bipartite nuclear localization signals (NLSs) in cargoes [35]. The members of the karyopherin-β protein family vary in their cargo specificity and directionality of transport, regulating cargo import (importins or nuclear-import receptors [NIRs]), export (exportins or nuclear-export receptors) or transport in both directions (biportins or bidirectional receptors) [36]. Among the 20 mammalian karyopherin-β family proteins, ten are classified as importins (KPNB1/IPO1, TNPO1/KPNB2, TNPO2/IPO3, IPO4, IPO5, IPO7, IPO8, IPO9, IPO11 and TNPO3/IPO12), five as exportins (XPO1/CRM1, XPO2/CAS, XPOT, XPO5 and XPO6) and three as biportins (IPO13, XPO4 and XPO7), whereas the functions of RanBP6 and RanBP17 are yet to be determined (for a detailed nomenclature see Table 1). Heterodimers of importin-α family members with importin-β1/KPNB1 mediate the bulk import of cargo with a classical NLS, whereas importin-β2/transportin-1/TNPO1 mediates the import of cargo with a proline-tyrosine (PY)-type NLS (Fig. 1). The export of proteins with a nuclear export signal (NES) depends on exportin-1/XPO1 [37]. Other modes of cargo binding via linear or folded protein domains or RNA structures recognized by karyopherin-β proteins are less well understood [36].

**Table 1:** Summary of the NTR family members and their NLS-dependent and -independent chaperone activity

NTR	Synonyms	Full name	Reported chaperone and/or disaggregase activity
*Karyopherin-α family: α-importins*			
**KPNA1**	NPI-1, RCH2	Importin-α5	
**KPNA2**	RCH1	Importin-α1	• Disrupt oligomerization of TDP-43 N-terminal domain [38] • Prevent nuclear SENP2 association with cytoplasmic membranes [39]
**KPNA3**	QIP2	Importin-α4	
**KPNA4**	QIP-1	Importin-α3	
**KPNA5**		Importin-α6	
**KPNA6**		Importin-α7	
**KPNA7**		Importin-α8	
*Karyopherin-β family: β-importins, exportins, biportins*			
* β-importins*			
**KPNB1**	IPO1, Kapβ1, NTF97	Karyopherin-β1, Importin-β1	• Chaperone and disaggregate TDP-43: via its classical NLS in concert with importin-α [38, 40, 41], and its PrLD in concert with FG-Nups [42] • Chaperone and disaggregate FUS via its RGG regions [43, 44] • Suppress poly(GR) condensation in vitro [41] • Reduce cytoplasmic aggregation of FG-Nups: Nup62 [42], Nup107, Nup153 and yeast Nup49 [45, 46] • Suppress in vitro aggregation of basic proteins: rpL4, rpL6 and histone H1 (together with IPO7), rpL18a [47]
**TNPO1**	IPO2, Kapβ2, KPNB2, TRN, MIP1	Transportin-1, Karyopherin-β2	• Chaperone and disaggregate FUS: via its PY-NLS [40, 48–54] and its RGG regions [43, 44] • Chaperone and disaggregate other RBPs with a PY-NLS: TAF15, EWSR1, hnRNPA1 and hnRNPA2 [40] • Suppress poly(GR) condensation in vitro [41] • Prevent nucleoporin Nup153 association with cytoplasmic membranes [55] • Suppress phase separation of CIRBP via its RG/RGG region [56]
**TNPO2**	IPO3, KPNB2B	Transportin-2, Karyopherin-β2b	• Reduce cytoplasmic TDP-CTF aggregation [42]
**TNPO3**	IPO12, TRN-SR	Transportin-3, Transportin-SR	• Reduce cytoplasmic TDP-CTF aggregation [42] • Chaperone and disaggregate FUS via its RGG regions [43, 44] • Suppress phase separation of CIRBP via its RSY region [56]
**IPO4**	RanBP4	Importin-4	• Reduce cytoplasmic TDP-CTF aggregation [42] • Prevent cytoplasmic accumulation of yeast nucleoporin Pom33 [57] • Chaperone histone complex H3-H4-ASF1 [58] • Suppress in vitro aggregation of basic ribosomal protein rpS3a [47]
**IPO5**	KPNB3, Kapβ3, RanBP5	Importin-5	• Suppress in vitro aggregation of basic ribosomal protein rpL23a [47]
**IPO7**	RanBP7	Importin7	• Chaperone and disaggregate FUS via its RGG regions [43, 44] • Suppress in vitro aggregation of basic proteins: rpL4, rpL6 and histone H1 (together with IPO7), rpL23a [47]
**IPO8**	RanBP8	Importin-8	
**IPO9**	RanBP9	Importin-9	• Reduce cytoplasmic TDP-CTF aggregation [42] • Chaperone histone complex H2A-H2B [59] • Suppress in vitro aggregation of basic ribosomal proteins: rpL18a, rpS7 [47]
**IPO11**	RanBP11	Importin-11	
* Exportins*			
**XPO1**	CRM1	Exportin-1	• Reduce formation of cytoplasmic FG-Nup condensates [60]
**XPO2**	CAS, CSE1L	Exportin-2	
**XPOT**	XPO3	Exportin-t	
**XPO5**	RanBP21	Exportin-5	
**XPO6**	RanBP20	Exportin-6	
* Biportins*			
**IPO13**	RanBP13	Importin-13	• Reduce cytoplasmic aggregation of TDP-CTF and Nup62 [42] • Prevent Ubc9 from interacting with its cytoplasmic partners during nuclear import [61]
**XPO4**		Exportin-4	• Prevent nucleolar aggregation of cytoplasmic eIF5A [62]
**XPO7**	RanBP16	Exportin-7	
* Unknown transport function*			
**RanBP6**		Ran-binding protein 6	
**RanBP17**		Ran-binding protein 17	

*Abbreviations*: *ASF1* Anti-silencing function 1, *CAS* Cellular apoptosis susceptibility protein, *CIRBP* Cold-inducible RNA-binding protein, *CRM1* Chromosome region maintenance 1 protein, *CSE1L* Chromosome segregation 1-like protein, *eIF5A* Eukaryotic translation initiation factor 5A, *FG-Nup* phenylalanine and glycine-rich nucleoporin, *MIP1* M9 region interaction protein 1, *NLS* nuclear localization signal, *NPI-1* Nucleoprotein interactor 1, *NTF97* Nuclear transport factor p97, *NTR* nuclear transport receptor, *PrLD* prion-like domain, *QIP-1* Importin alpha Q1, *RBP* RNA-binding protein, *RCH1* RAG cohort protein 1, *RSY* arginine-serine-tyrosine, *SENP2* Sentrin-specific protease 2, *TDP-CTF* TDP-43 C-terminal fragment, *Ubc9* Ubiquitin conjugating enzyme 9

### Cargo recognition by NIRs: NLS-dependent and -independent mechanisms

**Fig. 2:**
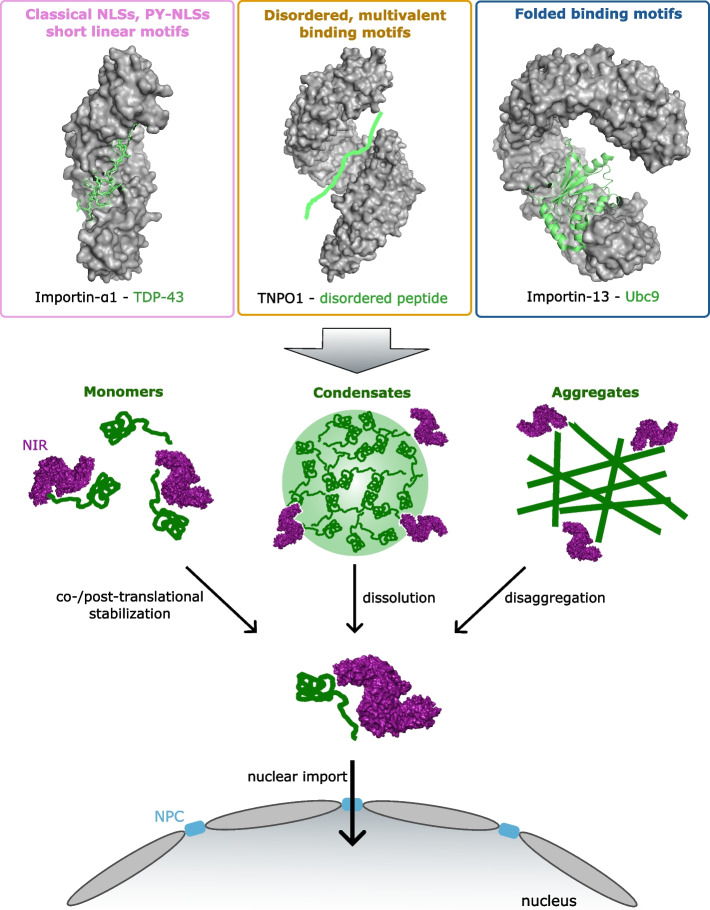
NLS-dependent and -independent binding and chaperoning of cargo by nuclear import receptors (NIRs). NIRs recognize their cargo by binding to linear and non-linear motifs. Linear motifs include classical or non-classical NLSs, disordered protein regions, and folded, three-dimensional motifs. This versatility of binding motifs allows NIRs to co- and post-translationally stabilize a plethora of monomeric proteins and dissolve condensates or disaggregate amyloid aggregates. Subsequently, monomeric cargo is guided across the NPC back into the nucleus. PDB-IDs: 7N9H, 2H4M, 2XWU

To ensure nuclear import of the appropriate proteins, NIRs specifically recognize and engage cargo via either folded domains or short, often linear motifs termed NLSs [36] (Fig. 2). NLSs can be grouped into classical monopartite or bipartite NLSs, and non-classical NLSs. Monopartite classical NLSs consist of a single cluster of 4–8 positively charged amino-acids with the consensus sequence K-K/R-X-K/R [64]. Conversely, bipartite classical NLSs are composed of two linker-connected clusters of 2–3 positively charged amino-acids with the consensus sequence R/K-X_10-12_-KRXK [64]. Proteins bearing classical NLSs are recognized and bound by importin-α in the cytoplasm followed by recruitment of importin-β1/KPNB1 [36]. This importin-β1/importin-α/cargo trimer can then be imported into the nucleus by temporarily breaking hydrophobic, intermolecular FG-Nup interactions that comprise the hydrogel barrier within the NPC [36, 37, 65–67].

TDP-43 bears a bipartite classical NLS (^82^KRK-MDETDASSAV-KVKR^98^), located between the TDP-43 N-terminal domain (NTD) and RNA-recognition motif 1 (RRM1), and can therefore be recognized and imported into the nucleus by the importin-α/β1 heterodimer (Fig. 1). Importin-α interacts with classical NLSs via a minor and major binding groove [68]. Detailed investigation of the structure of the importin-α1/TDP-43 complex has revealed that the interaction of arginine 83 in the TDP-43 NLS with the minor binding groove of importin-α1 is crucial and can be modulated by phosphorylation of threonine 88 in the linker region of the NLS [38]. Intriguingly, the TDP-43 NLS also harbors a poly(ADP-ribose) (PAR)-binding motif, which engages PAR. Thus, PAR may compete with importin-α for binding to the TDP-43 NLS, which may promote cytoplasmic mislocalization of TDP-43 under conditions where cytoplasmic PAR concentrations become elevated [6, 69–71].

PY-NLSs are non-classical, 20–30 amino-acid stretches containing N-terminal hydrophobic (ϕ) or positively charged ( +) residues and typically contain a PY motif at their C-terminal end. The consensus sequence is + /ϕ-X_n_-R/H/K-X_2-5_-PY [72]. PY-NLS-containing proteins can be recognized by Transportin-1 (TNPO1, Karyopherin β2), which imports cargo directly into the nucleus without the need for importin-α as adaptor protein [72]. FUS, other members of the FET protein family, and several hnRNPs (e.g., hnRNPA1, hnRNPA2, hnRNPD, hnRNPF, hnRNPM) contain a PY-NLS. The FUS PY-NLS (^501^GDRGGFGPGKMDSRGEHRQDRRERPY^526^) can be subdivided into three epitopes involved in the recognition by TNPO1. Epitope 1 (residues 508–511) contains hydrophobic/basic amino-acids, epitope 2 (residues 514–522) is arginine-rich and epitope 3 (residues 525–526) corresponds to the C-terminal PY motif [73]. TNPO1 interacts with the FUS PY-NLS at the C-terminal arch of the concave face of its super-helical structure by forming hydrophobic interactions at the N- and C-terminal region of the PY-NLS, and electrostatic interactions with a central α-helix in the FUS PY-NLS epitope 2 [73]. This binding pattern is similar but nuanced across a variety of TNPO1 cargo [74].

Many proteins which localize and function in the nucleus contain neither a classical nor a PY-type NLS, raising the question how and by which importin(s) these proteins are recognized and guided through the NPC. In silico approaches have identified novel consensus NLSs for IPO4, IPO5 [75] and IPO7 [76] with the help of molecular docking simulations based on interactions with known and suspected cargo (IPO4: LPPRS(G/P)P; IPO5: KP(K/Y)LV; IPO7: EKRKI(E/R)(K/L/R/S/T). These putative NLSs were validated in cells by measuring the subcellular localization of GFP-NLS fusion constructs upon siRNA-mediated knock-down of the corresponding NIR [75, 76].

There is increasing evidence that select importins can bind proteins via less well-defined sequences. Specifically, arginine-glycine-rich domains (RG/RGG) and serine-arginine-rich domains (SR) can be recognized and bound by different NIRs. Likewise, arginine-rich dipeptide repeats (R-DPRs, poly-GR and poly-PR) that are generated via repeat-associated non-ATG (RAN) translation from a disease-causing intronic hexanucleotide repeat expansion in the *C9orf72* locus in C9ALS/FTD [77], can also engage diverse NIRs [41, 78, 79]. Thus, there may be other undescribed features which enable selective nuclear import of specific cargo.

NIR binding to classical or PY-NLSs is characterized by high-affinity interactions with specific sequences. By contrast, RG/RGG- and RSY-based NIR-cargo interactions are of multivalent nature, often involving multiple, weak interaction sites [48, 56]. Interestingly, many disordered regions of phase-separating and amyloid-forming proteins contain RG/RGG-rich stretches, RS(Y)-rich stretches, or both, indicating that their localization and self-assembly could be regulated by NIRs. This conjecture is in line with the finding that several NIRs recognize and bind R-rich DPRs. In particular, KPNB1, IPO7 and importin-α3 interact with poly-GR and poly-PR, whereas TNPO1 and TNPO3 exhibit high-affinity binding with poly-GR [41, 79].

In addition to binding PY-NLSs, TNPO1 (Kapβ2) weakly recognizes RG/RGG-rich domains of cold-inducible RNA-binding protein (CIRBP) and FUS [40, 43, 48, 50, 56], as well as mutated (e.g., P525L) and truncated (e.g., R495X) FUS variants which lack the PY-NLS [44]. Arginine methylation in the RGG-rich domain adjacent to the FUS PY-NLS modulates TNPO1 binding: hypomethylated FUS permits TNPO1 binding and nuclear import, whereas arginine methylation weakens the TNPO1-FUS interaction [80]. This finding indicates that nuclear import of FUS can be finely tuned by post-translational modifications, representing a further layer of regulation of this process.

Another NIR which recognizes sequences alternative to classical or PY-NLSs is TNPO3, which exhibits high affinity for proteins with SR- and/or R(E/D)-rich regions [56, 81]. Typical cargo bearing such sequences are RBPs involved in RNA splicing, which are enriched in nuclear speckles and paraspeckles [82–85]. For instance, TNPO3 binds to an arginine-serine-tyrosine (RSY) rich region of CIRBP with nanomolar affinity. In synergy with TNPO1, this interaction is crucial to ensure the nuclear localization of CIRBP but also regulates CIRBP phase separation and recruitment to stress granules (SGs) [56].

In addition to NLSs defined by a consensus sequence, there is increasing evidence for cargo recognition based on folded motifs or a combination of both sequence and structure, as reviewed elsewhere [36]. Three-dimensional binding motifs are less well-defined and often involve larger globular domains. To identify and characterize such complex NIR-cargo interactions, rigorous structural analysis by X-ray crystallography or cryo-EM is important. Multiple NIRs recognize structured motifs, including importin-α3, importin-α6, KPNB1, IPO4, IPO5, IPO7, IPO8, IPO9, IPO11, IPO13, XPO4 and XPO7 [36]. However, detailed information of NIR-cargo interaction has been revealed for only a few examples. For instance, IPO4 binds the H3-H4-ASF1 histone-histone chaperone complex via three distinct binding interfaces. The N-terminal part of IPO4 recognizes the globular H3-H4 domain and an α-helical motif of H3, the C-terminal half of IPO4 binds the H3 N-terminal tail via multiple interactions across HEAT repeats 11–22 [58]. Importin-α3 interacts with Ran exchange factor RCC1 (regulator of chromosome condensation 1) via a different approach, by recognizing a combination of two short linear NLSs in addition to a large structured β-propeller domain. The latter not only contributes directly to the interaction with importin-α3, but also ensures cargo specificity by creating a distinct structural environment [86]. IPO13 is another example of a NIR recognizing folded domains, including those of Ubc9 and MAGO-Y14. Interestingly, these two cargoes interact with entirely different regions of IPO13. Ubc9 interacts mainly with the N-terminal arch of IPO13 (HEAT repeats 1–9), whereas MAGO-Y14 binds HEAT repeats 5–20 [61]. These examples highlight different binding modes that are exploited by NIRs to ensure cargo specificity in a manner which can extend beyond NLS recognition.

Despite these advances, there is still much to learn, as for many NIRs the corresponding NLS or folded recognition domain(s) remain unknown, and their respective cargoes are yet to be discovered. Recent studies have attempted to identify these cargoes and pinpoint their NLSs or common motifs that the respective NIR may bind. In affinity-based approaches, the NIR of interest is immobilized on a functionalized, stationary matrix followed by incubation with cell extracts. Bound substrates are then eluted and analyzed by immunoblotting, mass spectrometry (MS), or both. For instance, stable isotope labeling using amino-acids in cell culture (SILAC)-labeled cell extracts were used to identify import and export cargo that interact with immobilized IPO13 [87]. Unspecific binding was minimized by pre-incubation with Ubc9, a known IPO13 cargo, thereby only allowing the binding of putative cargo that can compete with the Ubc9-IPO13 interaction. Substrates that were found to bind only in presence of GTP-bound Ran were considered potential IPO13 export cargoes [87]. Similar experiments have identified cargoes of KPNB1, TNPO1, IPO5, IPO7 [88], TNPO2 [89], XPO1, XPO5 [90], XPO6 [91], and XPO7 [92]. As an elegant alternative to affinity-purification, a proximity proteomics method based on tagging 16 NTRs with the engineered promiscuous biotin ligase BirA* (BioID) was used to determine cargo specificity and systematically map cargo-NTR interactions in situ [93].

In a different approach (SILAC-Tp), non-labeled HeLa cell nuclear extracts, depleted of β-importins, were added to SILAC-labeled permeabilized HeLa cells. Cargo present in the non-labeled nuclear extract was then imported by NIRs into the nuclei of the isotope-labeled cells followed by analysis of the nuclear fraction by LC–MS/MS [94]. This approach did not require affinity-based isolation of cargo and led to the identification of cargoes of all ten importins and two biportins, including many that were previously unknown [82].

NIR-cargo binding can be validated using a spectrum of in vitro assays that quantify protein–protein interactions, including bead-halo assays, isothermal titration calorimetry (ITC), surface plasmon resonance (SPR), or nuclear magnetic resonance (NMR) experiments [56, 73, 95, 96]. In cells, NIR-cargo binding can be analyzed by measuring the efficiency of nuclear import of the cargo of interest by assessing its nucleocytoplasmic ratio by fluorescence microscopy, or by compartmental fractionation followed by western blotting [51, 87].

### Karyopherins dissolve the FG-Nup hydrogel to facilitate NCT

The formation of a selective size filter and the assembly and stability of the NPC may be facilitated by phase separation, the process of macromolecules de-mixing to form concentration-dependent phases with distinct compositions [27, 97–102]. This process is driven by multivalent interactions, including hydrophobic contacts, aromatic π-π contacts, and other types of contacts. Such interactions occur between patches enriched for certain amino-acids (such as phenylalanines and glycines) that are separated by linkers in the intrinsically disordered regions (IDRs) of FG-Nups [97]. Several specific sequence patterns of FG-repeats are known, including FxFG, GLFG, xxFG, PSFG, SAFG and VFG [97]. At high concentrations, Nup98 and other FG-Nups can spontaneously phase separate into liquid droplets and hydrogels with NPC-like permeability properties [19] and can transition into amyloid fibrils over time [45, 103, 104]. Aside from their canonical role in forming the permeability barrier for selective NCT, FG-Nups are highly mobile and can be found outside the NPC, where they may contribute to the formation of phase-separated membraneless organelles, such as SGs and processing bodies (P-bodies) [60, 105].

To transport cargo across the NPC, NTRs must navigate through the permeability barrier formed via hydrophobic interactions of FG-repeats in a hydrogel-like meshwork in the central channel of the NPC [97]. This activity requires dynamic and flexible conformational changes which allow NTRs to form rapid transient multivalent interactions with FG-Nups and disengage the hydrophobic interactions between FG-repeats, thus locally destabilizing the hydrogel to facilitate the transport of bulky cargo [66, 67]. Whereas the overall sequence similarity among β-karyopherins is low, they share a similar and evolutionary conserved architecture, where ~ 20 consecutive HEAT repeats are arranged in a flexible α-solenoidal structure [106]. Each HEAT repeat is a ~ 30–40 amino acid-long motif composed of two amphiphilic α-helices A and B linked by a short loop [107]. These A- and B-helices are arranged in an anti-parallel fashion, with the hydrophobic residues facing each other to stabilize their orientation. A-helices form the outer convex surface and B-helices form the inner concave surface of the NTRs [107]. The amphiphilic nature of NTRs allows them to rapidly adapt to both hydrophobic and hydrophilic environments. Indeed, when the NTR reaches the permeability barrier of the NPC, it contacts the FG-Nups at its convex surface, while holding its cargo at its concave core [65, 67, 108]. NTRs simultaneously bind to multiple FG motifs which allows them to temporarily disengage hydrophobic Nup-Nup interactions and permeate the meshwork [66]. The surface properties of NTRs grant them the ability to transiently engage FG-Nups with rapid binding and unbinding kinetics, thus ensuring a high transport rate across the NPC while maintaining the solubility of their cargo [66]. Adaptively positioned hydrophobic residues, cysteines, histidines and arginines on the surface of NTRs facilitate passage across the NPC, whereas surface lysine, glutamic acid and aspartic acid residues impede passage across the NPC [109].

### Karyopherins act as chaperones that prevent and disaggregases that reverse cytoplasmic protein aggregation

Beyond their canonical role as transport receptors, several studies have described a novel molecular chaperone function of NTRs in preventing abnormal aggregation of their cargo in the nucleus and cytoplasm. Early studies proposed that some NIRs chaperone positively charged proteins in the cytoplasm [47], and subsequently were found to function as histone chaperones [58, 59]. Yeast NIRs Srp1/Kap95, Kap121 and Kap123 function as co-translational chaperones, shielding positively charged patches of nascent, partially unfolded, aggregation prone RBPs from non-specific and inappropriate interactions thereby preventing aggregation [110]. Thus, NTRs can maintain the structural and functional integrity of cargo before delivery to their final destination. In this way, NIRs may resemble signal-recognition particles that chaperone nascent polypeptides bearing a signal sequence [111, 112].

Several importins maintain the solubility, structural and functional integrity of aggregation-prone basic cargo proteins such as ribosomal proteins and histones by shielding them from unspecific binding and cytoplasmic aggregation with RNA before delivery into the nucleus [47]. For example, IPO4 and IPO9 act as chaperones by wrapping around H2A-H2B and H3-H4-ASF1, to protect these histone complexes from inappropriate non-nucleosomal interactions while escorting them into the nucleus [58, 59]. Importin-α1 prevents nuclear SUMO-specific isopeptidase SENP2 from binding to intracellular membranes in the cytoplasm [39], and IPO13 shields SUMO-E2 conjugating enzyme Ubc9 from binding to its partners during its nuclear import to prevent off-target SUMOylation [61].

NIRs also play an important role in modulating the properties of FG-Nups in the hydrogel-like permeability barrier in the central channel of the NPC [113–115]. NIRs inhibit the aberrant aggregation and amyloid formation of FG-Nups under crowded conditions [45], and also prevent inappropriate association of FG-Nups with cytoplasmic membranes prior to their delivery to the nuclear membrane [55, 57].

Exportins also exhibit chaperone activity. For example, XPO4 antagonizes undesired interactions of elongation factor eIF5A inside the nucleoli and carries it to the cytoplasm [62]. Moreover, functional XPO1/CRM1 depletion via RNAi or pharmacological inhibition leads to an increase in cytoplasmic Nup foci in *C. elegans* oocytes, suggesting a role in promoting Nup solubility [60].

## Cytoplasmic mislocalization and aberrant phase transition of karyopherin cargoes in ALS/FTD

Phase separation, often driven by low-complexity PrLDs, allows RBPs to rapidly self-associate into ribonucleoprotein granules such as SGs to exert their physiological functions in RNA processing [116–120]. However, aberrant phase transition of RBPs into solid aggregates has been hypothesized to play a central role in disease [6, 116, 121, 122]. The distinctive composition of low-complexity PrLDs enriched in uncharged polar amino-acids and glycines [123] renders these RBPs highly interactive and prone to undergo aberrant phase transition, during which dynamic condensates mature into hydrogels or stable solid-like fibrils [124, 125]. Moreover, disease-causing mutations in PrLDs can accelerate this transition [122, 126–129]. Thus, dysregulated phase separation can be the initial step in the formation of pathological aggregates, which are the hallmark of several devastating neurodegenerative diseases [1, 129]. However, in other contexts, cytoplasmic liquid condensates of TDP-43 can be toxic [130, 131], and solid phases of TDP-43 confer beneficial outcomes, such as the amyloid-like oligomeric TDP-43 assemblies or “myo-granules” that are formed during skeletal muscle regeneration [132]. Hence, context must be considered when defining a phase transition as aberrant or deleterious [6].

NTRs play an important role in maintaining the soluble state of their cargo prior to and during their passage across the NPC. This NTR activity seems to be reduced in neurodegenerative diseases where the NTR cargo can become vulnerable to changes in the surrounding milieu and eventually undergo a deleterious phase transition [1]. This process can result from reduced NCT efficiency, as occurs in aging, which causes an accumulation of deteriorated and misassembled NPCs and expression changes in certain factors regulating NCT [133–135]. In addition, pathological sequestration of cargo may drive a deleterious phase transition [136, 137]. ALS and FTD are characterized by the abnormal cytoplasmic accumulation and phase transition of nuclear RBPs into pathological inclusions [138–141]. These PrLD-containing RBPs, including FUS, TAF15, EWSR1, hnRNPA1, hnRNPA2, and TDP-43, are all NIR cargoes and can readily undergo phase separation [7, 142].

### FET proteins, hnRNPA1, and hnRNPA2

Heterogeneous nuclear ribonucleoproteins (hnRNPs), including FET proteins (FUS, EWSR1 and TAF15), hnRNPA1 and hnRNPA2, are RBPs that are primarily located in the nucleus where they are involved in different steps of RNA metabolism, including transcription, pre-mRNA splicing, and RNA transport [7, 143, 144]. FUS has mainly been linked to ALS/FTD through genetics, pathology, or both. FUS pathology is observed in ~ 4% of familial and ~ 1% of sporadic ALS (sALS) cases, and ~ 9% of FTD cases [7, 145]. TAF15 and EWSR1 are connected to FTD-FUS and rare ALS cases [7, 146–148]. hnRNPA1 occurs as two isoforms, the more abundant isoform A and the less abundant isoform B which contains 52 additional amino acids in its PrLD [149, 150]. hnRNPA2 is the most abundant splicing isoform expressed from the *HNRNPA2B1* gene and best studied in the context of ALS. Other isoforms include hnRNPB1, A2b and B1b [127, 151]. Less than 1% of familial and sporadic ALS cases are associated with hnRNPA1 and hnRNPA2 pathology [127, 144, 152]. However, different members of the hnRNP family are associated with a variety of degenerative diseases including multisystem proteinopathy (MSP, hnRNPA1/A2), hereditary motor neuropathy (hnRNPA1), oculopharyngeal muscular dystrophy (hnRNPA2), spinal muscular atrophy (hnRNPG/Q/M/A1/R), Alzheimer’s disease (AD) (hnRNPA1/C/Q), ALS/FTD (hnRNPH/F/A1/A2/A3/E2/D/G/I/L/Q/R), multiple sclerosis (hnRNPA1/H), congenital myasthenic syndrome (hnRNPH/L), and fragile X-associated tremor/ataxia syndrome (hnRNPA2) [7, 143, 152, 153].

FET proteins, hnRNPA1, and hnRNPA2 are depleted from the nucleus and mislocalized to the cytoplasm in stable inclusions in postmortem brain tissue of patients with some forms of familial and sporadic ALS/FTD [127, 146–148, 154, 155]. Thus, pathogenicity has been linked to a combination of nuclear loss-of function, often manifesting in defects in transcription, splicing, and non-coding RNA turn-over [118, 156–158], and toxic gain-of-function, represented by cytoplasmic aggregates that are deleterious to neurons [126, 146, 147, 150, 159–161]. In vitro, these RBPs form amyloid-like fibrils, likely driven by steric zipper and low-complexity aromatic-rich kinked segment (LARK) interactions [146, 147, 160–166]. Due to inherent limitations of immunohistochemistry studies in autopsy tissue discussed in more detail below, additional confirmatory studies based on validated antibodies and antibody-independent spatial proteomic approaches are necessary to determine the scope of RBP mislocalization beyond TDP-43 in sporadic ALS/FTD.

FUS, TAF15, and EWSR1 consist of an N-terminally disordered PrLD including a QSYG-rich and an RGG-rich region, as well as a C-terminal structured domain including an RRM, zinc finger domain, two or three RGG-rich domains and the PY-NLS [7] (Fig. 1). Disease-associated mutations are found throughout FUS, although pathogenicity has been mainly associated with those located at the C-terminal end [7, 167]. In particular, the substitution of FUS proline 525 with leucine (P525L) or tyrosine 526 with cysteine (Y526C), both of which disrupt the PY-NLS, cause a particularly aggressive form of juvenile ALS [168–170].

hnRNPA1 and hnRNPA2 share a similar domain architecture, containing two N-terminal RRMs and a C-terminal PrLD which includes the PY-NLS [7, 144]. Several mutations in *HNRNPA1* and *HNRNPA2B1* are connected to ALS and MSP [7, 127, 152, 153]. For example, the substitution of an aspartic acid with valine (D262V in hnRNPA1, D290V in hnRNPA2) leads to the loss of a repulsive negative charge in the fibril backbone combined with the gain of a hydrophobic amino-acid which in turn promotes fibrillization [7, 127, 162, 171]. The hnRNPA1 mutations P340A/S (P288A/S in the shorter isoform) substituting the critical proline residue in the PY-NLS are likely to weaken binding to TNPO1 and are equivalent to P525L in FUS [152, 172, 173]. Similarly, frameshift mutations in hnRNPA2 causing early-onset oculopharyngeal muscular dystrophy have been shown to disrupt binding to TNPO1 [153].

FET proteins, hnRNPA1, and hnRNPA2 are recruited to membraneless organelles in the nucleus (e.g. nucleolus, paraspeckles) and in the cytoplasm (e.g., SGs, RNA transport granules), but can also form amyloid-like fibrils [118, 142]. In addition, these RBPs phase separate in vitro and undergo a liquid-to-solid transition over time, modulated by ALS-associated mutations and post-translational modifications [49, 116, 122, 174, 175]. Several disease-associated amino-acid substitutions in hnRNPA1 and hnRNPA2 significantly alter SG formation and enhance phase separation and fibrillization in vitro and in vivo [116, 127, 152]. Likewise, disease-linked mutations in TAF15 and EWSR1 accelerate protein aggregation [116, 127, 146, 147, 152].

### TDP-43

TDP-43 is also an RBP that plays a critical role in regulating RNA metabolism [6, 120, 176]. TDP-43 predominantly resides in the nucleus but can shuttle to the cytoplasm to regulate RNA stability and SG assembly and dynamics [177, 178]. Mobile TDP-43-containing granules are also present in axons, with a potential role in regulating mRNA localization and local translation [179–183]. TDP-43 proteinopathy is characterized by the abnormal redistribution of TDP-43 from the nucleus into the cytoplasm, leading to both loss and toxic gain-of-function phenotypes: loss of nuclear TDP-43 causes mis-splicing events and cryptic exon inclusion in key neuronal genes such as *STMN2* and *UNC13A* [184–187], whereas accumulated cytoplasmic TDP-43 forms detergent-insoluble aggregates that sequester proteins involved in various cellular pathways [10, 188–191]. Pathologically aggregated TDP-43 is ubiquitinated, hyperphosphorylated, acetylated, and partially cleaved, with several C-terminal fragments (CTFs) with molecular weights of ~ 18–27 kDa forming a major component of phosphorylated TDP-43 (pTDP-43) inclusions in the brain of ALS/FTD patients [138–140, 192]. ALS and FTD are considered primary TDP-43 proteinopathies, where ~ 97% and ~ 45% of ALS and FTD cases, respectively, are characterized by TDP-43 pathology [145]. Mutations in the gene encoding TDP-43 (*TARDBP*) can directly cause disease [193–195]. Another age-related neurodegenerative disease defined by TDP-43 proteinopathy is limbic-predominant age-related TDP-43 encephalopathy (LATE) [196]. Pathological TDP-43 inclusions also frequently occur as a comorbid pathology in several neurodegenerative diseases defined by other distinct pathological protein aggregates, such as Alzheimer's disease, Huntington's disease, Lewy body disease, and progressive supranuclear palsy [197–199].

TDP-43 is comprised of a well-folded NTD, a classical bipartite NLS, two RNA-recognition motifs (RRM1 and 2) and a C-terminal intrinsically disordered PrLD [7] (Fig. 1). TDP-43 phase separation is largely driven by its PrLD and by the oligomerization of its NTD, which can be modulated by post-translational modifications and different biomolecules [120, 128, 129, 200–203]. Phosphorylation of the NTD inhibits not only phase separation of TDP-43, but also its pre-mRNA splicing activity as a functional consequence [201]. By contrast, RNA binding to TDP-43 can promote phase separation, while maintaining the liquid-like properties and solubility of TDP-43 condensates [204, 205]. The nuclear abundance of RNAs, specifically GU-rich transcripts, dictates nuclear TDP-43 localization and solubility [204, 206]. RNA can also prevent pathological TDP-43 oligomerization, phase separation, and aggregation [129, 207]. TDP-43 phase separation is also promoted by PAR binding to the TDP-43 NLS [69].

In the nucleus, TDP-43 localizes to membraneless organelles such as paraspeckles and Cajal bodies [117, 208, 209], and phase separates into droplets with symmetrical liquid spherical shells and liquid cores, termed anisosomes [210]. Anisosome formation can be triggered by ALS/FTD disease-causing mutations or post-translational acetylation of lysine residues in the RRMs of TDP-43 (K145 and K192), which impair RNA interaction with TDP-43. Also present in these nuclear foci are Hsp70 chaperones, which prevent TDP-43 conversion into pathological gel-like TDP-43 aggregates [210, 211].

Nuclear import of TDP-43 is driven the importin-α/β1 complex, where the NLS of TDP-43 is recognized and bound by several importin-α family members [212]. Importin-α1 preferentially associates with the minor site of the NLS, and post-translational modifications in proximity of that site abrogate this interaction and lead to cytoplasmic accumulation of TDP-43 [38]. By regulating its subcellular localization, the importin-α/β1 import complex also governs TDP-43 splicing function, phase separation and solubility [38, 40, 41].

TDP-43 was initially thought to harbor an NES that regulates its nuclear egress [213], but further mechanistic studies found that this NES is not functional and that TDP-43 can passively exit the nucleus independently of exportin XPO1 [214, 215] and when it is not bound by nuclear GU-rich RNAs [206]. Selective inhibitors of nuclear export (SINE) that target XPO1 have a protective effect in cellular and animal models of ALS [216–218]. However, these SINEs do not restore TDP-43 nuclear localization; instead, they might inhibit the export of other nuclear proteins, thus reestablishing the nucleocytoplasmic balance by counteracting impaired nuclear import.

The pathomechanisms underlying the cytoplasmic mislocalization and pathological phase transition of TDP-43, as well as the order in which these events occur, are still poorly understood. Reduced RNA binding can cause mislocalization and aberrant phase transitions of TDP-43 [129, 205, 206, 219]. ALS/FTD-causing mutations within or adjacent to the RRMs (P112H, D169G, K181E, K263E) reduce RNA binding and increase aggregation propensity of TDP-43 [220–222], similar to introducing lysine acetylation mimics in RRM1 (K136, K145) and RRM2 (K192) [210, 219, 223, 224]. Likewise, ALS-linked mutations in the PrLD of TDP-43 accelerate aberrant phase transitions [129, 225]. Another possible initiating factor is that during aging and repeated periods of environmental stress or disease, TDP-43 condenses in cytoplasmic SGs, which over time may transition into pathological gel- or solid-like irreversible inclusions, providing a potential mechanism that integrates environmental with genetic factors [69, 116]. Indeed, chronic optogenetic induction of SGs causes them to evolve into pathological TDP-43 inclusions [226].

Nevertheless, there appear to be additional SG-independent routes to the formation of cytoplasmic, pathological TDP-43 inclusions [69, 70, 117, 129, 130, 227, 228]. For example, early TDP-43 aggregates induced by seeding of preformed fibrils or overexpression of TDP-43 CTFs appear highly enriched in Nups but not SG components [130, 216]. SGs also contain numerous Nups and NTRs, potentially disrupting NCT [136]. However, stress-induced NCT deficits can occur independently of SG formation [229]. Thus, the relationship between SGs and Nup-enriched cytoplasmic foci and their role in recruiting TDP-43 in the ALS/FTD disease process are still unclear. Together with studies on C9ALS/FTD pathology, these findings suggest an important role for NCT defects in the disease process [78, 230–235].

## NPC and karyopherin abnormalities in ALS/FTD

The identification of multiple NCT factors as disease suppressors in yeast and fly models of C9ALS/FTD pathology [232–234, 236, 237] led to the investigation of these pathways in ALS/FTD. Indeed, accumulating evidence implicates NCT defects in the pathogenesis of ALS/FTD and other late-onset neurodegenerative diseases [8–11, 238, 239]. This evidence raises an important question about causality: do NCT defects cause proteinopathies, or are NCT defects the consequence of cytoplasmic protein aggregates? The discovery that TDP-43 mislocalization into cytoplasmic aggregates can itself trigger NCT defects suggests the existence of a positive feedback loop, whereby cytoplasmic aggregation pathology and NCT defects feed into each other, potentially becoming self-sufficient of the initiating trigger [216, 240]. Loss of TDP-43 nuclear function leads to altered processing of *KPNB1* mRNA, which could also exacerbate cytoplasmic mislocalization and aggregation of TDP-43 [120]. This model is supported by the identification of rare missense variants and frameshift mutations in the *NUP50* gene encoding nucleoporin 50 as a risk factor for ALS [241]. Nup50 has functions beyond NCT, including chromatin biology and gene expression [242]. The downstream implications of these polymorphisms are unclear, and it will be interesting to see whether NUP50 variants are linked to TDP-43 pathology in ALS/FTD.

The mislocalization and aggregation of NTR and Nup components of the NCT machinery in various ALS/FTD mouse models and in human postmortem central nervous system (CNS) tissue suggests that defects in these pathways are potential causes and consequences of disease (summarized in Table 2). It should be noted that immunohistochemistry studies in postmortem human tissue can differ widely with regard to unambiguous staining results, the specificity of antibodies used, whether lipofuscin autofluorescence has been quenched, the number of cases analyzed, and whether blinded quantification was performed, making it difficult to interpret these data. To account for these disparities, information on the cases and relevant analysis parameters of each reference are included in Table 2. Additional thorough and quantitative neuropathological studies are necessary to confirm the extent of NTR and Nup mislocalization in ALS/FTD.

**Table 2:** Summary of NTR and nucleoporin defects in ALS/FTLD-TDP brain and spinal cord autopsy tissue

Protein	Tissue	Reported defects	Clinical cases	Analysis type
***α-importins***				
** KPNA2**	FTLD-TDP temporal cortex	Decreased nuclear and cytoplasmic labelling due to reduced protein levels [212]	11 FTLD-TDP (3F, 8 M), 9 controls (5F, 4 M)	Qualitative and blinded semi-quantitative
	sALS spinal cord	Enhanced cytoplasmic staining due to increased protein levels [212]	10 sALS (6F, 4 M), 10 controls (3F, 7 M)	Qualitative and blinded semi-quantitative
** KPNA4**	C9ALS/FTLD and sFTLD-TDP frontal cortex	Decreased nuclear labelling, colocalization with pTDP-43 inclusions, weak colocalization with poly-GA, GP, and GR inclusions [240]	8 C9ALS/FTLD, sporadic FTLD-TDP, 8 controls	Blinded qualitative
** KPNA6**	sALS spinal cord	Decreased protein levels [212]	10 sALS (6F, 4 M), 10 controls (3F, 7 M)	Qualitative and blinded semi-quantitative
***β-importins***				
** KPNB1**	sALS spinal cord	Decreased nuclear and increased cytoplasmic reactivity of KPNB1 [243]	7 sALS (3F, 4 M), 6 controls (3F, 3 M)	Qualitative
	ALS spinal cord AHCs	Decreased nuclear labelling and cytoplasmic accumulation [244]	6 ALS (3F, 3 M), 6 controls (5 M, 1F)	Quantitative
	C9ALS spinal cord	Depletion from the nucleus and nuclear envelope and cytoplasmic accumulation [245]	11 C9ALS cases (4F, 7 M), 8 non-C9ALS (4F, 4 M), 3 controls (1F, 2 M, 2AD/DLB),	Qualitative
	sALS spinal cord	Depletion from the nucleus or irregular nuclear KPNB1 staining in cells with TDP-43-positive inclusions [246]	Number of cases or controls unclear	Qualitative
	sALS spinal cord	Disrupted nuclear staining and increased cytoplasmic KPNB1 expression in cells with TDP-43 inclusions [247]	6 sALS (5 M, 1F), 7 controls (6 M, 1F)	Qualitative and quantitative
	sALS, TDP-ALS, C9ALS, sFTLD-TDP, c9FTLD-TDP hippocampus, motor cortex and spinal cord	Loss from the nucleus and sequestration into pTDP-43 inclusions [42]	2 C9ALS (1F, 1 M), 2 sALS (1F, 1 M), 2 SOD1-ALS (1F, 1 M), 1 FUS-ALS (1F, 0 M), 1 FUS-ALS (0F, 1 M), 1 TARDP-ALS (0F, 1 M), 2 C9FTLD-A (1F, 1 M), 2 C9FTLD-B (0F, 2 M), 4 controls (0F, 4 M)	Qualitative
** TNPO1**	FTLD-FUS frontal cortex, hippocampus, medulla, and spinal cord	Nuclear and cytoplasmic aggregation, colocalization with FUS inclusions [248]	6 NIFID (5F, 1 M), 7 aFTLD-U (3F, 4 M), 3 MSA, 3 CBD, 3 MND, 3 AD, 3 PD, 3 PSP, 3 PiD, 1 FTLD-TDP-1, 3 FTLD-TDP-2, 1 FTLD-TDP-3, 3 controls	Qualitative
	FTLD-FUS hippocampus and cerebral white matter	Nuclear and cytoplasmic aggregation [249]	17 aFTLD-U (9F, 8 M), 8 BIBD (3F, 5 M), 4 NIFID (3F, 1 M), 6 ALS-FUS (4F, 2 M), 6 FTLD-TDP (2 Type A, 2 Type B, 2 Type C), 9 FTLD-Tau (2 PiD, 2 CBD, 5 PSP), 2 CHMP2B-FTLD, 3 FTLD-ni, 2 sALS-TDP, 2 SOD1-ALS, 2 AD, 4 DLB, 8 PD, 2 MSA, 2 HD, 3 SCA (1 SCA-1, 2 SCA-3), 1 NIBD, 4 controls	Qualitative
	FTLD-FUS hippocampus and temporal cortex	Cytoplasmic aggregation [250]	5 aFTLD-U (1F, 4 M), 2 NIFID (2F, 0 M), 1 BIBD (0F, 1 M)	Qualitative and semi-quantitative
	FTLD-FUS frontal cortex	Cytoplasmic aggregation, colocalization with FUS inclusions [251]	3 FUS-ALS (2F, 1 M), 3 C9-ALS (0F, 3 M), 7 sALS (5 M, 2F), 3 FTLD- FUS (3F, 0 M), 6 FTLD-TDP (3F, 3 M), 1 AD, 7 controls	Qualitative
***Exportins***				
** XPO2**	FTLD-TDP temporal cortex	Decreased nuclear and cytoplasmic labelling due to reduced protein levels [212]	11 FTLD-TDP (3F, 8 M), 9 controls (5F, 4 M)	Qualitative and blinded semi-quantitative
***Nucleoporins***				
** NUP50**	sALS lumbar spinal cord	Nuclear aggregation [252]	3 ALS, 1 control	Qualitative
** NUP54**	sALS spinal cord	Colocalization with pTDP-43 inclusions [253]	2 C9ALS/FTLD (1F, 1 M), 2 sALS (1F, 1 M), 1 sALS/FTLD (0F, 1 M), 2 FTLD-FUS (0F, 2 M), 1 control (0F, 1 M)	Qualitative and semi-quantitative
** NUP62**	sALS and SOD1-ALS spinal cord	Irregular nuclear contour staining [243]	7 sALS (3F, 4 M), 4 SOD1-fALS (0F, 4 M), 6 controls (3F, 3 M)	Semi-quantitative
	ALS spinal cord AHCs	Depletion from the nuclear envelope [244]	6 ALS (3F, 3 M), 6 controls (5 M, 1F)	Quantitative
	sALS spinal cord AHCs	Nuclear depletion in ADAR2- and TDP-43-negative cells, disrupted nuclear staining in cells with cytoplasmic TDP-43 inclusions [246]	No indication of number of cases or controls	Qualitative
	sALS spinal cord	Depletion or disruption of Nup62 staining in cells with mislocalized TDP-43 [247]	6 sALS (5 M, 1F), 7 controls(6 M, 1F)	Qualitative and quantitative
	sALS medial temporal cortex and spinal cord; C9ALS/FTLD hippocampus and spinal cord	Nuclear disruption, colocalization with pTDP-43 inclusions [253]	2 C9ALS/FTLD (1F, 1 M), 2 sALS (1F, 1 M), 1 sALS/FTLD (0F, 1 M), 2 FTLD-FUS (0F, 2 M), 1 control (0F, 1 M)	Qualitative and semi-quantitative
	sALS, TDP-ALS, C9ALS, sFTLD-TDP, c9FTLD-TDP hippocampus, motor cortex and spinal cord	Loss from the nuclear envelope and sequestration into pTDP-43 inclusions [42]	2 C9ALS (1F, 1 M), 2 sALS (1F, 1 M), 2 SOD1-ALS (1F, 1 M), 1 FUS-ALS (1F, 0 M), 1 FUS-ALS (0F, 1 M), 1 TARDP-ALS (0F, 1 M), 2 C9FTLD-A (1F, 1 M), 2 C9FTLD-B (0F, 2 M), 4 controls (0F, 4 M)	Qualitative
** NUP88**	sALS and SOD1-ALS lumbar spinal cord	Irregular nuclear contour staining [243]	7 sALS (3F, 4 M), 6 controls (3F, 3 M)	Qualitative
** NUP98**	C9ALS/FTLD hippocampus and medial temporal cortex	Colocalization with p62 inclusions [253]	2 C9ALS/FTLD (1F, 1 M), 2 sALS (1F, 1 M), 1 sALS/FTLD (0F, 1 M), 2 FTLD-FUS (0F, 2 M), 1 control (0F, 1 M)	Qualitative and semi-quantitative
** NUP107**	C9ALS motor cortex	Abnormal perinuclear aggregation [232]	6 C9ALS (4F, 2 M), 5 non-C9ALS (3F, 2 M), 2 C9ALS/FTLD (0F, 2 M), 6 controls (3F, 3 M)	Qualitative and semi-quantitative
** NUP153**	sALS and SOD1-ALS lumbar spinal cord	Irregular nuclear contour staining [243]	7 sALS (3F, 4 M), 6 controls (3F, 3 M)	Qualitative
** NUP205**	C9ALS motor cortex	Abnormal nuclear staining [232]	6 C9ALS (4F, 2 M), 5 non-C9ALS (3F, 2 M), 2 C9ALS/FTLD (0F, 2 M), 6 controls (3F, 3 M)	Qualitative and semi-quantitative
	sALS lumbar spinal cord	Colocalization with TDP-43 inclusions [252]	3 ALS, 1 control	Qualitative
	sALS and TDP-ALS motor cortex; C9ALS motor cortex; sALS, TDP-ALS and C9ALS frontal cortex; TDP-ALS motor cortex and hippocampus	Loss of nuclear envelope immunoreactivity, formation of large cytoplasmic inclusions, and colocalization with pTDP-43 aggregates [216]	2 TARDP-ALS (0F, 2 M), 7 C9ALS (4F, 3 M), 1 SOD1-ALS (1F, 0 M), 15 sALS (6F, 9 M), 10 controls (4F, 6 M)	Qualitative
** GP210**	sALS lumbar spinal cord	Increased staining in nuclear envelope and cytoplasm [252]	3 ALS, 1 control	Qualitative

*Abbreviations*: *AHC* anterior horn cell, *sALS* sporadic ALS, *pTDP-43* phospho-TDP-43, *AD* Alzheimer’s disease, *DLB* dementia with Lewy bodies, *NIFID* neuronal intermediate filament inclusion disease, *MSA* multiple system atrophy, *CBD* corticobasal degeneration, *MND* motor neuron disease, *PD* Parkinson’s disease, *PSP* progressive supranuclear palsy, *PiD* Pick’s disease, *BIBD* basophilic inclusion body disease, *NIBD* neurofilament inclusion body disease, *SCA* spinocerebellar ataxia, *HD* Huntington’s disease

NTR irregularities were discovered in the G93A SOD1-Tg-mouse line where KPNB1 was found to co-aggregate with ubiquitin in anterior horn cells (AHCs) of the lumbar spinal cords, with decreasing nuclear-to-cytoplasmic ratios of KPNB1 and importin-α1, which worsened with the disease course [254]. Nup62, Nup88, and Nup153 staining irregularities were also described in SOD1-Tg-mice, with KPNB1 nuclear clearance and irregular Nup62 staining observed in SOD1-ALS and sALS patient spinal cord tissue [243]. Since these initial studies, various defects in the distribution of NTRs and Nups were reported in FUS, TDP-43, C9orf72, and sporadic ALS/FTD models and patient tissue (Table 2).

NTR and FG-Nup abnormalities in FTLD-FUS and ALS-FUS post-mortem brain tissue center around TNPO1 [73, 255]. In FTLD-FUS brain tissue, the predominantly nuclear protein TNPO1 accumulates in cytoplasmic aggregates that contain all members of the FET protein family [248–251]. This finding is in contrast to familial ALS-FUS, where ALS-linked FUS mutations clustered around the PY-NLS weaken FUS-TNPO1 binding [50, 73, 255] and neither TNPO1 nor EWSR1 or TAF15 are recruited into FUS-positive inclusions [249]. Aberrant FUS-Nup interactions are linked to NCT defects, but co-aggregation of FUS with Nups has not been observed in ALS/FTD [42, 253, 256].

Prominent NTR and FG-Nup irregularities have also been described in ALS/FTD post-mortem tissue with TDP-43 pathology. KPNB1 exhibits decreased nuclear staining and increased cytoplasmic localization in sALS spinal cord tissue [243–247]. KPNB1 colocalizes with pTDP-43 inclusions in sALS-TDP, FTLD-TDP and C9ALS/FTD, but not in ALS-SOD1 or ALS-FUS patient tissue [42]. XPO2/CAS and importin-α1 protein levels are decreased in FTLD-TDP patient brain tissue, whereas in sALS-TDP spinal cord tissue, total levels of importin-α1 and -α7 are increased and decreased, respectively [9, 212]. Importin-α3 is mislocalized from the nucleus, and partially colocalizes with pTDP-43 in C9ALS/FTD and sFTD cases with TDP-43 pathology [240]. These findings indicate a more general disruption of NTRs in TDP-43 proteinopathies, beyond its transporter KPNB1.

Irregular nuclear staining of Nup50, Nup62, Nup88 and Nup153 was first described in sALS cases [243, 244, 246, 247, 252]. Nup62 accumulates in pTDP-43 aggregates in sALS-TDP, FTLD-TDP and C9FTLD-TDP [42, 253]. Nup205 and FG-Nups Nup54 and Nup98 are also sequestered in TDP-43 inclusions in ALS/FTD patient tissue [216, 232, 252, 253]. Loss of specific Nups has also been observed in ALS/FTD patient-derived iPSCs and isolated nuclei [230, 235]. These changes were preceded by an increase of CHMP7, a mediator of NPC quality control, in nuclei of C9orf72 and sALS patient iPSC-derived spinal neurons, suggesting a role for CHMP7-mediated Nup defects as a potential pathomechanism for ALS [235]. Intriguingly, CHMP7 may form a grommet to preserve nuclear integrity [257], indicating that increased CHMP7 in the nucleus may be an initial response to prevent nuclear pore defects in ALS.

## Therapeutic potential of NIRs countering aberrant phase transitions in ALS/FTD

Although the connection between aberrant RBP condensates and ALS/FTD offers many opportunities to discover new drug targets, their heterogeneity in composition, location, and physical properties makes developing novel therapeutics a challenging undertaking. Exploiting molecular chaperones as therapeutic agents to counteract protein misfolding and aggregation has been a longstanding, attractive idea since they evolved to control proteostasis [2, 14, 18, 258–260]. Indeed, a multitude of chaperones, often acting synergistically in chaperone networks, can prevent and reverse formation of amyloid-like structures including protein fibrils and oligomeric species [2, 18].

In ALS/FTD, however, where pathological cytoplasmic mislocalization and aggregation of physiologically nuclear proteins are hallmarks of disease, a potential therapeutic agent must be able to not only solubilize aberrant, toxic protein assemblies but also promote nuclear import of the resulting monomers and/or newly synthesized proteins to restore proteostasis [2, 18]. In this context, several members of the family of human NIRs naturally possess remarkably effective chaperoning activity while restoring physiological nuclear localization of ALS/FTD-associated RBPs [14, 17, 40–42, 48–51] (summarized in Table 1). Due to this dual functionality, NIRs are attractive, novel drug candidates with highly promising therapeutic potential for ALS/FTD and related disorders [14, 17].

### NLS-dependent chaperone function

The heterodimer importin-α/β1 and the β-importin TNPO1 effectively counteract aberrant self-assembly of RBPs containing disordered PrLDs, and classical and PY-NLSs, respectively [14, 17, 40, 41, 48–54]. TNPO1 has been demonstrated to prevent and reverse the formation of liquid-like droplets, hydrogels and fibrils, formed by FUS, TAF15 and EWSR1, as well as several disease-associated variants, in vitro [40, 48, 51]. TNPO1 failed to effectively counteract the self-assembly of variants lacking the PY-NLS, but retains modest activity at high concentrations, indicating additional interaction sites on cargo [40, 44, 48–50]. In FUS, the majority of ALS-causing missense mutations and truncations are clustered around the C-terminal PY-NLS, and K510 acetylation disrupts TNPO1 binding, resulting in the mislocalization and aggregation of FUS in the cytoplasm [261]. This indicates that unhindered access to the PY-NLS is crucial to ensure stabilization and chaperoning of soluble FUS by TNPO1. At the molecular level, small-angle X-ray scattering experiments have revealed that FUS adopts a more compact structure in complex with TNPO1 than when alone in solution [48]. Indeed, TNPO1-mediated disaggregation of FUS fibrils results in soluble TNPO1-FUS complexes that are competent for nuclear transport, stabilizing the FUS monomer against re-aggregation [40]. Importantly, in addition to counteracting aberrant FUS self-assembly in vitro, TNPO1 also combats pathological hallmarks of ALS/FTD in vivo [40, 51]. Overexpression of TNPO1 dissolves cytoplasmic FUS foci in yeast and mammalian cell models, rescues FUS cytoplasmic mislocalization and recruitment into SGs, and buffers against FUS-associated cytotoxicity [40, 51]. TNPO1 successfully rescues FUS-induced rough eye phenotype and neurodegeneration in *Drosophila,* proving a therapeutic effect in metazoa [40].

In addition to its effect on proteins of the FET family, TNPO1 effectively prevents and reverses hnRNPA1 and hnRNPA2 self-assembly in vitro [40]. TNPO1 also mitigates muscle degeneration caused by disease-linked hnRNPA2 variants in *Drosophila* [40]. In hnRNPA1 and hnRNPA2, the PY-NLS, which is crucial for interaction with TNPO1, is buried within the fibril core [40, 164, 262, 263]. Consequently, TNPO1 disaggregates hnRNPA1 and hnRNPA2 fibrils more slowly than FET protein fibrils [40]. Comparison of structures of the hnRNPA1 PY-NLS in complex with TNPO1 and in an hnRNPA1 PrLD fibril suggests that binding of TNPO1 to hnRNPA1 via its PY-NLS (residues 263–289 in the short isoform) promotes an extended conformation of the PY-NLS, which impedes fibril formation [164].

In contrast to FUS, EWSR1, TAF15, hnRNPA1, and hnRNPA2, TDP-43 contains a classical bipartite NLS instead of a PY-NLS. Consequently, TNPO1 fails to counteract aberrant TDP-43 self-assembly, whereas the importin-α/β1 heterodimer which binds classical NLSs prevents seeded and unseeded fibril formation of wild-type TDP-43 and ALS-associated variant TDP-43^Q331K^ in vitro [40]. Importin-α/β1 heterodimers also effectively prevent TDP-43 phase separation [41]. By contrast, importin-α/β1 complex was ineffective against fibrils formed by TDP-43 variants lacking the classical NLS and failed to prevent and reverse the self-assembly of proteins containing a PY-NLS [40]. The interaction of importin-α/β1 with TDP-43 sterically interferes with NTD-mediated TDP-43 oligomerization, which may contribute to the observed inhibition of TDP-43 self-assembly in presence of the importin-α/β1 heterodimer [13, 38]. Similar to FUS, introducing an acetylation mimic in the NLS (K84Q) increases TDP-43 cytoplasmic mislocalization, presumably by disrupting interactions with importin-α-associated KPNB1.

The vast majority (> 40 variants) of ALS/FTD-linked mutations are located in the PrLD of TDP-43 [7, 264, 265] (Fig. 1). Curiously, however, a C-terminal frameshift variant of TDP-43 in the PrLD with an increased propensity to form solid fibrils instead of liquid-like condensates, causes rimmed vacuole myopathy but not ALS/FTD [266]. ALS/FTD-disease causing mutations in the RRMs of TDP-43 may increase its aggregation propensity by inhibiting RNA binding [221, 267]. While one potential ALS-associated variant in the NLS (A90V) was found to drive cytoplasmic localization of TDP-43 [268], this variant is also found in the healthy population and has only very minor effects on protein solubility and aggregation in vitro [269, 270].

### NLS-independent chaperone function

Recent evidence demonstrates that canonical NLSs in RBPs are not the only binding sites for NIRs to exert their nuclear import, chaperoning and disaggregation activities. Karyopherin-β family importins can directly bind NLSs, folded domains, or a combination of both to recognize and engage their cargo [36]. Several NIRs, including TNPO3 and IPO13, can bind dozens of cargo proteins with no defined consensus NLS [95]. KPNB1 and other importins were shown to chaperone RNA- and DNA-binding proteins with exposed basic domains but also FG-Nups, as discussed below [47, 59].

NLS-independent cargo binding and chaperoning by NIRs is only beginning to be understood, and likely has therapeutic potential with respect to antagonizing aberrant phase transitions. In particular, RGG- and RSY-rich domains as well as R-rich DPRs undergo phase separation and aggregation and can be recognized by several NIRs [41, 43]. While arginines drive phase separation by complex coacervation or by promoting intermolecular cation-π interactions, π-π interactions [271], polar amino-acids such as serines and tyrosines are found in LARKS, which are short peptide sequences that mediate the formation of labile amyloid-like fibrils [165]. Therefore, NIRs could also be exploited therapeutically to target aberrant assemblies of protein fragments and variants lacking NLSs, or with mutations therein.

#### FUS

The ALS-associated variant FUS^R495X^ is a truncated form that lacks the PY-NLS but is still partially detected in the nucleus [44]. Interestingly, TNPO1 reduced phase separation and fibrillization of FUS^R495X^ when added at high concentrations, although the effect was less pronounced compared to FUS^WT^ [40, 44]. Upon engaging the PY-NLS, TNPO1 makes secondary weak and dynamic interactions with other portions of FUS, including its N-terminal low-complexity PrLD, disordered RGG domains, and with residues 164–500 which contain folded and unfolded domains [44, 48] [48, 50] (Fig. 1). FUS^WT^ uses its high affinity PY-NLS to bind TNPO1, whereas FUS^R495X^ that is missing the PY-NLS mostly relies on its RGG motifs for TNPO1 binding [44]. In the context of FUS^WT^ fibril disaggregation, TNPO1 likely engages the PY-NLS that is exposed on the surface of the FUS fibril, followed by secondary contacts with the FUS PrLD to dissociate intermolecular contacts that hold the fibril together [40, 48, 51]. In the latter scenario, TNPO1 inhibits FUS^R495X^ phase separation by disrupting cation-π or π-π interactions between arginine residues in the RGG/RG domains and tyrosine residues in the FUS PrLD [40, 44, 48–50]. Importantly, arginine methylation can modulate TNPO1 binding and chaperone activity, with TNPO1 exhibiting higher affinity for hypomethylated FUS [80, 272]. This finding explains why TNPO1 strongly accumulates in aggregates containing hypomethylated FUS in FTLD-FUS cases [248, 273, 274]. Moreover, TNPO1 weakly engages the RRM and ZnF domain of FUS to cause it to eject bound RNA [48, 50]. Since RNA concentration modulates FUS phase separation [49, 50, 52–54, 207], this adds another layer of regulation for TNPO1 by potentially disrupting FUS-RNA interaction.

Intriguingly, multiple additional NIRs, including KPNB1, TNPO3 and IPO7, prevent and reverse FUS self-assembly by binding RGG domains in FUS [43, 44]. These importins could be therapeutically exploited to antagonize aberrant aggregation of ALS-associated variants of FUS which lack the C-terminal PY-NLS or weaken the TNPO1-PY-NLS interaction [7, 275]. Both TNPO1 and TNPO3 also suppress phase separation of CIRBP by binding its RGG motifs [56]. Similar to FUS, arginine methylation of the RG/RGG domains in CIRBP reduces TNPO1 and TNPO3 binding and thus CIRBP nuclear translocation [56]. TNPO3 also transports arginine-rich nuclear-speckle protein SRRM2 [82, 276], further highlighting the importance of arginine residues in mediating the interaction between NIRs and their cargo.

#### TDP-43

KPNB1 and other β-type importins mitigate aggregation, mislocalization and neurotoxicity of TDP-43 variants lacking its classical NLS across different models of TDP-43 proteinopathy [42]. KPNB1 reduces cytoplasmic aggregation of truncated TDP-CTFs and full-length TDP-43 constructs where the classical NLS was mutated to abrogate binding of importin-α, suggesting that KPNB1 might engage the TDP-43 PrLD directly or indirectly to antagonize TDP-43 aggregation. Mapping experiments indicated that KPNB1 can be immunoprecipitated from cell lysates with the PrLD of TDP-43, as well as its RRM2 domain, although RRM2 is not required for the effects of KPNB1 on TDP-43 [42]. While the splicing isoform short-TDP-43 (sTDP), which lacks the PrLD but still harbors an intact NLS [277], can form a complex with KPNB1, its cytoplasmic aggregation was only mildly reduced by KPNB1 expression, suggesting that sTDP aggregates via a different mechanism than TDP-CTFs, and is less effectively antagonized by KPNB1 [42].

Mutating the Nup-interacting site in the active N-terminal fragment of KPNB1 (HEAT repeats 1–8) abolished FG-Nup binding and its ability to reduce TDP-CTF aggregation [42]. Together with previous findings that FG-Nups associate with the PrLD of TDP-43 [216], this finding suggests that FG-Nups could mediate the interaction between KPNB1 and TDP-43 PrLD in the cytoplasm, and possibly the KPNB1-dependent reduction of TDP-CTF aggregation. Nup62, an FG-Nup that promotes TDP-43 proteinopathy [278], and KPNB1 colocalize with pTDP-43-positive inclusions in postmortem CNS tissue of ALS/FTD patients [42, 253], and Nup62 overexpression increased the recruitment of KPNB1 to cytoplasmic TDP-CTF aggregates [42]. Phenylalanine residues throughout the PrLD promote TDP-43 droplet formation and fibril formation [42, 279, 280], and KPNB1 has been proposed to bind to FG-like repeats in the TDP-43 PrLD, either directly or indirectly via FG-Nups [13]. However, removing phenylalanine residues in TDP-43 PrLD did not affect its co-aggregation with Nup62, while surprisingly, introducing additional phenylalanine residues in the TDP-43 PrLD abrogated this co-aggregation [42].

The finding that detergent-insoluble cytoplasmic sTDP aggregates that do not colocalize with Nup62 and Nup98 due to the absence of the PrLD, were not strongly reduced by KPNB1, indicated that FG-Nups might play a role in the reduction of TDP-CTF aggregation by KPNB1 [42]. Turbidity assays using purified components indicated that KPNB1 can partially prevent and reverse formation of TDP-CTF condensates [42]. Moreover, HEAT repeats 1–9 of KPNB1 also exerted this activity, which was reduced by mutating the N-terminal Nup-interacting site [42].

KPNB1 expression also relocated cytoplasmic TDP-43 lacking an NLS back to the nucleus in an FG-Nup-dependent manner [42]. Importantly, KPNB1 reduced cytoplasmic TDP-43-induced toxicity in neuronal cells and in fly models of TDP-43 proteinopathy [42]. A genetic modifier screen in flies expressing human TDP-43 carrying an ALS-causing mutation [281] established that reduced levels of Ketel, the fly ortholog of KPNB1, enhances TDP-43 toxicity in vivo [42]. Overexpression of Ketel reduces retinal degeneration, TDP-43 hyperphosphorylation, motor defects, and death in flies [42]. IPO13, which shows robust activity towards TDP-CTF aggregates in neuronal cells, also rescues neuron eye degeneration in TDP-43^ΔNLS^ flies, further confirming that multiple NIRs can reduce TDP-43 proteinopathy [42]. It will be interesting to investigate whether a similar mitigation of TDP-43-mediated neurodegeneration can be achieved by specific NIRs in additional models of TDP-43 proteinopathy, and if these measures restore TDP-43 function in vitro and in vivo.

#### C9DPRs

Several independent genetic modifier screens in yeast and fly models of C9ALS converged on components of the NCT machinery, implicating both C9RNA foci and DPR pathology in causing NCT defects [232–234]. Multiple NIRs were identified as modulators of R-DPR toxicity in C9ALS/FTD models [10]. Overexpression of TNPO1, TNPO3, IPO9, IPO11 and importin-α4 mitigated poly-PR toxicity in yeast [233], and KPNB1 knockdown in poly-PR and poly-GR-expressing flies worsened rough eye degeneration [237, 282]. However, most of these studies failed to uncover whether NIRs directly target R-DPRs or rather correct G_4_C_2_-induced neuronal defects. Upregulation of NIRs in yeast did not change the levels or distribution of poly-PR aggregates, suggesting that NIRs abrogated toxicity by reestablishing normal NCT via indirect mechanisms [233].

Further studies showed that R-DPRs can sequester NIRs and may impede their function of preventing RBP phase transition in ALS/FTD. In cells, R-DPR inclusions were found to be positive for NIRs KPNB1 and TNPO1, FG-Nups, but also several ALS-related RBPs including TDP-43, hnRNPA1 and Matrin-3 [78, 137]. Poly-GR and poly-PR can affect the phase separation of multiple RBPs and disturb the assembly and dynamics of membraneless organelles [283]. R-DPRs can render TDP-43 and multiple NIRs insoluble and disrupt nuclear import of TDP-43 [41, 137]*.* Of note, high levels of KPNB1 or TNPO1 suppressed poly-GR condensation in vitro and prevented poly-GR from stimulating TDP-43 aggregation [41]. KPNB1 prevents TDP-43 sequestration within cytoplasmic poly-GR aggregates in an FG-Nup-dependent manner, but without eliminating poly-GR aggregates themselves [42]. Importin-α3 and -α4 were also found to reroute cytoplasmic TDP-43 back to the nucleus in a neuronal model of poly-glycine-alanine (poly-GA) pathology [284]. Importantly, TNPO1 mitigates poly-GR-mediated toxicity in cell culture [285]. These findings suggest that elevated levels of NIRs can be a promising therapeutic opportunity to counter R-DPR toxicity in C9ALS/FTD.

#### Nucleoporins

FG-Nups are intrinsically disordered proteins that are prone to phase separate and form condensates in the cytoplasm during NPC biogenesis [60, 97, 286]. Transient post-mitotic cytoplasmic PML (promyelocytic leukemia) bodies were found to incorporate FG-Nups in a KPNB1-dependent fashion, suggesting that NIRs may function as chaperones and assembly factors for FG-Nups at the NPC permeability barrier and in cytoplasmic assemblies [287]. A variety of chaperones, including the MLF2-Hsp70 complex, DNAJB6, and KPNB1, protect against the sequestration of FG-Nups by aberrant cytoplasmic NPC-like assemblies that induce NCT defects [21, 46, 288].

Evidence for the relevance of FG-Nup condensates in disease was reported in the context of early-onset DYT1 dystonia, a neurological movement disorder caused by a mutation in the ATPase TorsinA [21]. TorsinA deficiency compromises NCT and proteostasis due to the formation of aberrant cytoplasmic nuclear envelope blebs, instead of mature NPCs. These blebs stain positive for FG-Nups, DNAJB6 and Hsp70 proteins [21, 288]. Under physiological conditions, Nup62 and other FG-Nups also form mobile cytoplasmic foci that are positive for DNAJB6 [288] and KPNB1 [287]. Interestingly, recombinant FG-Nups aggregate under crowding conditions, which can be inhibited by DNAJB6 [288] or KPNB1 [19, 45, 46]. KPNB1-positive cytoplasmic granules were also found in cortical and motor neurons of the mouse CNS, where loss of C9orf72 increased their abundance and changed their composition by disrupting association with FG-Nups [289]. It will be interesting to see how this C9orf72 loss-of-function phenotype may contribute to the TDP-43 pathology observed in C9ALS/FTD disease models and patients.

Aside from KPNB1, several other β-importins, including IPO4, IPO7 and IPO9, were previously found to function as cytoplasmic chaperones for ribosomal and histone proteins [47, 59] and to reduce formation of TDP-CTF aggregates [42]. Interestingly, β-importins, but not exportins, also reduce cytoplasmic aggregation of Nup62 [42], suggesting that KPNB1 and other NIRs can antagonize intermolecular interactions that enable formation of cytoplasmic FG-Nup condensates. Exportins strongly colocalized with Nup62 aggregates but did not affect their size and number [42]. However, an increase in cytoplasmic Nup foci in *C. elegans* oocytes after reducing XPO1/CRM1 activity via RNAi or an inhibitor suggests that XPO1 may also play a role in regulating FG-Nup solubility [60]. These Nup foci are enriched for FG-Nups, including orthologs of human Nup62 and Nup98, and may represent toxic condensates that are actively repressed in healthy cells.

### Importin variants and fragments with optimized activity

**Fig. 3:**
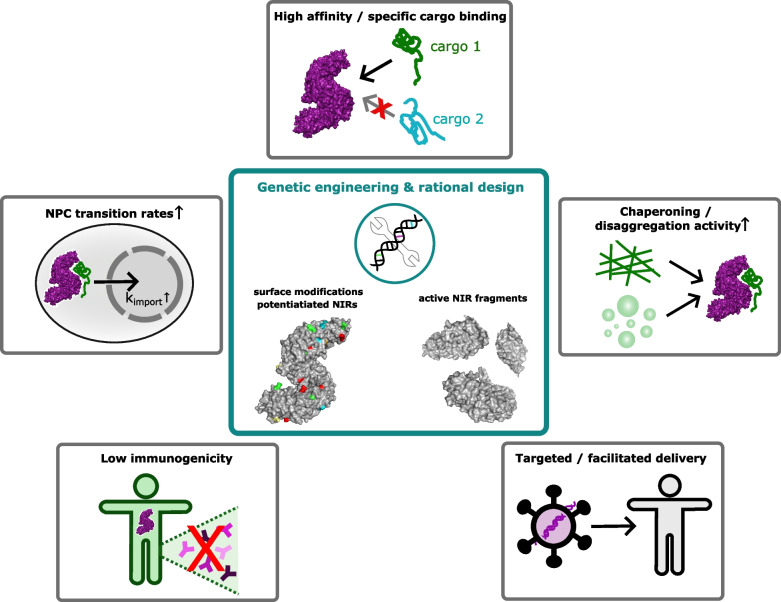
Designing and engineering NIR-based drug candidates with optimized activity and suitable properties for delivery. Starting from naturally occurring, wild-type NIRs, specific cargo binding, improved chaperoning activity and increased NPC transition rates can be achieved by generating variants with altered cargo-binding affinities and amino acid substitutions at the NIR surface. Smaller, active NIR fragments will facilitate NIR gene delivery via AAVs and could exhibit lower immunogenicity. PDB-ID: 2H4M

The discovery that NIRs counter deleterious phase transitions of RBPs has led to the idea that NIRs can be utilized for therapeutic applications to restore RBP homeostasis and mitigate neurodegeneration [14, 15, 17, 18, 42, 142]. It is unclear why TNPO1 accumulates in FUS-positive inclusions in FTLD-FUS rather than preventing FUS aggregation, since TNPO1 can efficiently reduce phase separation of hypomethylated FUS in vitro and in cells [40, 49]. The same question also applies to KPNB1 which accumulates in TDP-43-positive inclusions in ALS/FTD, while it can efficiently reduce TDP-43 pathology in models of TDP-43 proteinopathy [42]. It appears that in late-onset neurodegenerative diseases the activity and protein levels of endogenous NIRs are insufficient to prevent pathological phase transitions. This failure raises the question of whether naturally occurring NIRs can be modified to develop potentiated variants that outperform the wild-type chaperoning activity and nuclear import efficiency [17, 290, 291] (Fig. 3).

To be applied as effective therapeutics, it may be beneficial for potential NIR-based drug candidates to modulate certain properties. Among these are cargo specificity and affinity, high passage rate through the NPC, low molecular weight, and the ability to effectively target proteins in monomeric and oligomeric states, as well as mesoscopic phase-separated condensates and solid-like aggregates. Optimal NIR-cargo interactions must be reversible to ensure cargo dissociation by Ran-GTP in the nucleoplasm. To improve the recognition of cargo bearing disease-causing mutations in the NLS, it will be important to gain detailed structural information on the NIR-cargo binding sites if it is currently not available. Increasing the NIR-cargo binding affinity could be achieved by introducing mutations within the cargo binding site of NIRs, or by developing small molecules that stabilize the NIR-cargo interaction [14]. In addition to rationally designed variants, amino-acid substitutions that result in potentiated chaperone activity can be identified via a fully unbiased approach by combining error-prone PCR with yeast disease models [290]. Thus, a library of variants of a chaperone of interest, or subdomains thereof, could be generated and tested for its efficiency to antagonize aggregation and cytotoxicity of a disease protein of interest co-expressed in yeast or human cells. This strategy has been successfully applied to identify potentiated variants of the yeast protein disaggregase Hsp104 [292–296], including potentiated variants with enhanced substrate specificity [297], and could be also used to identify NIRs with potentiated activity.

In addition to optimal cargo recognition, high import efficiency of the desired cargo into the nucleus may be a beneficial feature of a potential NIR-based therapeutic protein candidate, provided that endogenous NIRs are unable to perform this task. Of note, the surface amino-acid composition of engineered GFP and KPNB1 proteins greatly affects their passage rates through the NPC [109]. In particular, the substitution of lysine with arginine residues at the protein surface accelerates NPC passage rates due to cation-π and increased π-π interactions with FG-Nups, whereas lysine residues can only engage via cation-π interactions [271]. Although rapid passage across the NPC is a naturally given property of NIRs, the substitution of superficial lysines with arginines could represent a strategy to further improve NIR-cargo NPC transition rates.

For therapeutic application of biologics, it would be highly beneficial if the drug candidate has a low molecular weight and small size. This property has been explored in depth with respect to therapeutic antibodies that often exhibit a molecular weight well above ~ 100 kDa, comparable to full-length human NIRs [298]. Using nanobodies or antibody fragments instead of full-size antibodies provides several pharmacodynamic and -kinetic advantages including facilitated recombinant production, increased stability, improved penetration of barriers in the body and enhanced affinity and specificity for the target [299, 300]. A similar approach could also be beneficial for NIR-based biologics. Encouragingly, shorter fragments of TNPO1 and KPNB1 are sufficient to chaperone FUS and TDP-CTF assemblies, respectively, and to mediate subsequent nuclear import [42, 51]. Specifically, a C-terminal fragment of TNPO1 containing only HEAT repeats 8–20 prevents and reverses FUS aggregation and phase separation in vitro, solubilizes cytoplasmic FUS foci and restores nuclear localization of FUS in yeast and human cells, and suppresses FUS-associated toxicity in yeast [51]. This construct is the smallest fragment that fully preserves all mapped TNPO1-FUS PY-NLS interactions [73]. Similarly, an N-terminal fragment of KPNB1 containing HEAT repeats 1–8 is necessary and sufficient to reduce TDP-CTF aggregation and toxicity in cellular models of TDP-43 proteinopathy [42].

A future challenge is the translation of a NIR-based drug candidate into a clinically applicable therapeutic [2, 17, 51, 292–294]. To achieve this goal, it will be crucial to identify suitable methods ensuring tissue specific, highly efficient delivery. One approach could be the use of adeno-associated virus (AAV) particles carrying the gene for the modified potentiated NIR. To minimize the size of the packaged gene construct (maximal packaged genome size ~ 5 kb [301]), potentiated smaller NIR fragments will be beneficial. In addition to AAV-based approaches, lipid nanoparticles (LNPs) could be another strategy to package and deliver NIR mRNA to be translated in situ [302–304]. It is also conceivable to develop small molecules that bind to the endogenously expressed target NIR to mimic the effect of potentiating amino-acid substitutions [14]. Such strategies could be particularly effective to strengthen single-site NIR-cargo interactions but might be less successful for multi-site interactions.

Another challenge is the minimization of immunogenicity. This unfavorable immune response could be evoked by the therapeutic NIR itself or by the vehicle used for delivery (Fig. 3) and induce the formation of anti-drug or AAV-neutralizing antibodies [305–307]. Chemical modifications, including PEGylation or glycosylation of the biologics themselves [308], AAV capsid modifications and shielding [309–312], as well as using synthetic vectors, including LNPs, for gene or mRNA delivery [313] may reduce immunogenicity and could be potential strategies also for successful NIR-based therapies.

## Perspectives

NIRs act as potent protective modifiers of prion-like RBP pathology by reversing toxic protein aggregation and cytoplasmic mislocalization, thus mitigating neurodegeneration caused by nuclear loss-of-function and cytoplasmic gain-of-toxicity [14, 17, 42]. Of particular interest are recent findings that NIRs can chaperone their cargo via both NLS-dependent and -independent mechanisms, thus expanding the repertoire of aggregation-prone targets for NIRs. Indeed, NIRs could also find applications in treating tauopathies and polyglutamine disorders [314–316].

Interestingly, FG-Nups co-aggregate with many neurodegenerative disease-causing proteins, suggesting that beyond ALS/FTD, NIRs can target a variety of aggregation-prone proteins via cytoplasmic FG-Nups. In Huntington’s disease (HD), Nup62 and other Nups co-aggregate with cytoplasmic mutant Huntingtin in HD patients, iPSC-derived neurons, and mouse models [317, 318]. In addition, tau pathology drives cytoplasmic aggregation of Nup62 and Nup98 in AD brain tissue [319].

In closing, we propose that NIRs can modulate deleterious phase separation of disease-linked RBPs and other proteins through both NLS-dependent and -independent chaperone mechanisms. It will be important to investigate the neuroprotective effect of NIRs in in vivo models of proteinopathies. Increasing protein expression or activity of NIRs, either pharmacologically or by using engineered potentiated variants, may be used therapeutically to reverse pathological phase transition of ALS/FTD-related RBPs and other FG-Nup-associated disease proteins, and ultimately alleviate neurodegeneration. A recent cryo-EM study has shown that while all FET proteins formed insoluble aggregates with TNPO1 in the brain tissue of four FTLD-FUS cases, only TAF15 was observed to form filaments [320]. It will be interesting to see whether NIRs can be designed as therapeutic tools targeting TAF15 proteinopathy in “FTLD-FET” [147].

## Data Availability

Not applicable.

## Abbreviations

AAV: Adeno-associated virus
AD: Alzheimer’s disease
AHC: Anterior horn cell
ALS: Amyotrophic lateral sclerosis
ARM: Armadillo
CIRBP: Cold-inducible RNA-binding protein
CNS: Central nervous system
C9DPR: Dipeptide repeats expressed from aberrant C9orf72
C9ALS: ALS associated with aberrant C9orf72 hexanucleotide repeats
CTF: C-terminal fragment
cryo-EM: Cryogenic electron microscopy
EWS: Ewing's sarcoma protein
FET: Fused in sarcoma (FUS), Ewing’s Sarcoma Protein 1 (EWSR1), TATA-binding protein-associated factor 15 (TAF15)
FG-Nup: Phenylalanine and glycine-rich nucleoporin
FTD: Frontotemporal dementia
FTLD: Frontotemporal lobar degeneration
FUS: Fused in sarcoma
HD: Huntington’s disease
HEAT: Huntingtin, elongation factor 3 (EF3), protein phosphatase 2A (PP2A), and signaling kinase TOR1
hnRNP: Heterogeneous nuclear ribonucleoprotein
IDR: Intrinsically disordered region
IPO: Importin
iPSC: Induced pluripotent stem cell
ITC: Isothermal titration calorimetry
kDa: Kilodalton
KPNB1: Karyopherin-β1
LARK: Low-complexity aromatic-rich kinked segment
LATE: Limbic-predominant age-related TDP-43 encephalopathy
LC–MS/MS: Liquid chromatography with tandem mass spectrometry
LNP: Lipid nanoparticle
MSP: Multisystem proteinopathy
NCT: Nucleocytoplasmic transport
NER: Nuclear export receptor
NES: Nuclear export signal
NIR: Nuclear import receptor
NIS: Nucleoporin-interacting site
NLS: Nuclear localization signal
NMR: Nuclear magnetic resonance
NPC: Nuclear pore complex
NTD: N-terminal domain of TDP-43
NTR: Nuclear transport receptor
PAR: Poly(ADP-ribose)
P-bodies: Processing bodies
PrLD: Prion-like domain
pTDP-43: Phospho-TDP-43
PY-NLS: Proline-tyrosine nuclear localization signal
RAN: Repeat-associated non-ATG
RanBP: Ran-binding protein
RBP: RNA-binding protein
R-DPR: Arginine-rich dipeptide repeat
RGG: Arginine-glycine-glycine-rich motif
RRM: RNA-recognition motif
RSY: Arginine-serine-tyrosine-rich domain
sALS: Sporadic ALS
SG: Stress granule
SILAC-Tp: Stable isotope labeling by amino-acids in cell culture in vitro transport
SINE: Selective inhibitor of nuclear export
SOD1: Superoxide Dismutase 1
SR: Serine-arginine-rich domain
sTDP: Short-TDP-43 isoform
TAF15: TATA-binding protein-associated factor 15
TDP-43: TAR DNA-binding protein-43
TDP-CTF: TDP-43 C-terminal fragment
Tg: Transgenic
TNPO: Transportin
Ubc9: Ubiquitin conjugating enzyme 9
WT: Wild-type
XPO: Exportin
